# Digital Health Readiness and Medicare Primary Care Spending: Nationwide County-Level Observational Analysis

**DOI:** 10.2196/105190

**Published:** 2026-09-17

**Authors:** Zhaoqiang Zhou, John Geracitano, Baiming Zou, Saif Khairat

**Affiliations:** 1 School of Data and Information Sciences, University of North Carolina at Chapel Hill, Chapel Hill, NC, United States; 2 Carolina Health Informatics Program, University of North Carolina at Chapel Hill, 428 Carrington Hall, CB 7460, Chapel Hill, NC, 27514, United States, 1 919-843-5413; 3 Department of Biostatistics, Gillings School of Global Public Health, University of North Carolina at Chapel Hill, Chapel Hill, NC, United States; 4 School of Nursing, University of North Carolina at Chapel Hill, Chapel Hill, NC, United States; 5 The Cecil G. Sheps Center for Health Services Research, University of North Carolina at Chapel Hill, Chapel Hill, NC, United States

**Keywords:** digital health, primary care, health informatics, population health, health economics, health policy

## Abstract

**Background:**

Digital health technologies are increasingly promoted as mechanisms to improve care coordination, enhance access, and reduce health care costs. However, whether community-level digitalization is associated with lower primary care spending in Medicare-participating safety net settings remains unclear, particularly in federally qualified health centers (FQHCs) and rural health clinics (RHCs).

**Objective:**

This study examined the association between county-level digitalization and Medicare primary care spending for FQHC and RHC services across US counties. Specifically, we assessed whether higher digitalization was associated with lower spending, whether observed associations reflected persistent differences between counties or changes within counties over time, and how digitalization interacted with health care access and socioeconomic conditions.

**Methods:**

We conducted a county-level observational study of 2993 US counties from 2017 to 2023. Digitalization was measured using the Digital Health Index (DHI) digitalization subindex, and the outcome was geographically adjusted for per capita Medicare spending on FQHC and RHC services. A hybrid within-between panel model was used to distinguish between-county from within-county associations. Additional analyses included threshold models, digitalization trajectory analyses, and interaction models. Using 2023 cross-sectional data, multivariable regression and Extreme Gradient Boosting models with Shapley additive explanations were applied to evaluate the relative contributions of digitalization, health care access, and socioeconomic conditions to spending variation.

**Results:**

Higher county-level digitalization was associated with lower Medicare primary care spending. In longitudinal analyses, the association was driven primarily by persistent between-county differences (β=–67.62, 95% CI −73.51 to −61.74; *P*<.001), whereas within-county changes in digitalization were not significantly associated with spending (β=–2.72, 95% CI −9.10 to 3.67; *P*=.40). Compared with counties in the lowest digitalization tertile, spending was substantially lower among counties in the highest tertile (β=–165.05, 95% CI −179.74 to −150.36; *P*<.001). Counties characterized by early and sustained digital development also demonstrated significantly lower spending (β=–123.34, 95% CI −138.24 to −108.43; *P*<.001). In a separate 2023 cross-sectional analysis of the complete DHI, health care access exhibited the strongest inverse association with spending (β=–71.13, 95% CI −81.20 to −61.07; *P*<.001), followed by digitalization (β=–48.48, 95% CI −59.75 to −37.21; *P*<.001), whereas socioeconomic conditions were positively associated with spending (β=22.01, 95% CI 13.12-30.89; *P*<.001). Among the 3 DHI dimensions examined, Shapley additive explanations analyses identified health care access and digitalization as the most informative predictors of county-level spending variation.

**Conclusions:**

Higher county-level digitalization was associated with lower Medicare primary care spending in FQHC and RHC settings. However, the association reflected long-standing structural differences across counties rather than short-term changes in digital capacity. These findings suggest that digitalization functions as a component of broader health system capacity and may contribute to improved primary care efficiency when supported by sustained investment, adequate health care access, and favorable local conditions.

## Introduction

Primary care spending in Medicare-participating safety net settings is a persistent policy pressure point, particularly for medically underserved and rural populations served by federally qualified health centers (FQHCs) and rural health clinics (RHCs) [1-4]. Digital health technologies, including electronic health records, telehealth, and health information exchange, are being deployed across these settings with the expectation that expanded digital infrastructure will improve care coordination, reduce inefficiency, and lower costs [5,6]. However, because FQHCs and RHCs operate under payment models distinct from specialty and hospital-based services, Medicare spending in these settings reflects not only patient needs but also structural features of local primary care systems, including access, organizational capacity, and the degree to which digital infrastructure is in place and functional [7-9]. Whether digitalization actually reduces spending in these contexts or, instead, expands use in populations with high unmet need remains an open and consequential question.

Existing evidence on digitalization and health care spending has focused mainly on overall expenditures or hospital-based use, often treating digitalization as a short-term or marginal exposure rather than a structural feature of place [3,10]. Primary care spending in Medicare-participating FQHCs and RHCs remains substantially understudied despite the fact that workforce shortages, access barriers, and limited organizational capacity in these settings directly shape care delivery and spending patterns [11]. No county-level study has separated the contribution of persistent structural differences in digitalization from within-county changes over time, leaving open whether observed associations reflect enduring characteristics of place or short-term dynamics [12,13]. How digitalization interacts with health care access and socioeconomic conditions to shape primary care spending is similarly unexamined [14,15].

Conceptually, digitalization may reduce primary care spending by enhancing care coordination, reducing duplicative services, and enabling remote monitoring and population health management [16,17]. At the same time, digital infrastructure may expand access in underserved populations with high unmet need, increasing use and per capita spending [18,19]. These mechanisms are potentially offsetting, and the direction of association may depend on the structural context in which digitalization is deployed.

To address these gaps, we leveraged the Digital Health Index (DHI), a composite measure capturing multidimensional digital health capacity across digitalization, health care access, and socioeconomic conditions [20]. Using the DHI, we conducted a county-level study of the association between digitalization and per capita Medicare payments for FQHC and RHC services, a measure of primary care spending within the Medicare system [21]. We examined (1) whether higher digitalization was associated with lower per capita Medicare spending for FQHC and RHC services, (2) whether this association reflected persistent cross-county differences or within-county changes over time, and (3) how health care access and socioeconomic conditions interact with digitalization in shaping spending patterns.

## Methods

### Study Design

We conducted a county-level observational study to examine the association between digitalization and primary care spending in the United States. The analytic strategy followed a multistage design integrating longitudinal, cross-sectional, and distribution-focused approaches. Given the observational, county-level nature of the study, results should be interpreted as associative rather than causal.

First, we analyzed longitudinal panel data from 2017 to 2023 to characterize the temporal structure of the association between county-level digitalization and Medicare spending for FQHC and RHC services. A primary objective was to distinguish persistent between-county differences from within-county changes over time.

Second, informed by the longitudinal findings, we conducted complementary analyses, including nonlinear threshold models, digitalization trajectory analyses, and effect modification models, to assess whether the association between digitalization and primary care spending differed across structural contexts.

Third, we focused on the latest available 2023 data to perform a decomposition analysis aimed at disentangling the relative and joint contributions of digitalization, health care access, and socioeconomic conditions to cross-county variations in primary care spending. Machine learning–based heterogeneity and tertile-specific analyses were further applied to examine contextual and distributional variation.

### Data Sources

Primary care spending at the county level was measured using Medicare geographic variation data, specifically per capita Medicare payment for FQHC and RHC services, adjusted for geographic differences in payment rates [21]. The denominator was the eligible original Medicare beneficiary population in each county. The outcome was limited to Medicare payments for services delivered in these settings and did not represent total Medicare primary care spending across all health care settings. Spending values were analyzed as reported in the source data and were not additionally adjusted for general inflation. Year fixed effects accounted for common annual price growth and spending-level changes in the longitudinal models, whereas the reported dollar magnitudes remain expressed in nominal terms. This measure was available annually from 2017 to 2023 and served as the primary outcome in all analyses. Geographic adjustment improved comparability in spending across counties.

Digital health readiness was measured using the DHI, a validated, published composite measure of multidimensional capacity relevant to digital health [20,22]. The full DHI comprises 21 indicators across 3 subindexes: digitalization, health care access, and socioeconomic conditions. Indicators were oriented so that higher values represented more favorable conditions, combined using equal weights within each subindex, and rescaled to range from 0 to 1.

The digitalization subindex included measures of internet subscription, cellular data plans, device access, broadband coverage, and broadband upload and download speeds. The health care access subindex included vehicle access, medically underserved area designation, access to urgent care, and access to an emergency department. The socioeconomic conditions subindex included demographic composition, English-language proficiency, insurance coverage, public program enrollment, disability, poverty, food assistance, unemployment, and educational attainment. None of the 3 subindexes directly included FQHC or RHC availability, use, or Medicare FQHC or RHC spending.

For longitudinal analyses, we used the county-level digitalization subindex for 2017 to 2023 to capture temporal variation in digital infrastructure and capacity. Annual indicator values were percentile ranked using a common pooled reference distribution comprising all county-year observations from 2017 to 2023. Accordingly, annual scores were comparable across years and reflected each county’s digitalization relative to a consistent study period reference. For cross-sectional analyses, we used the full DHI in 2023, the latest year for which all 3 subindexes were available at the county level. Because the longitudinal and cross-sectional scores were generated within different scoring frameworks, their raw distributions are not directly comparable. All DHI measures were standardized and oriented so that higher values reflected more favorable conditions, thereby facilitating model estimation and interpretation.

Counties with available digitalization and Medicare FQHC and RHC spending data from 2017 to 2023 were included. County-year observations with missing exposure or outcome data were excluded without imputation, whereas reported zero-spending values were retained.

### Statistical Analysis

All DHI measures were standardized within their respective analytic samples before modeling; accordingly, the reported coefficients represent spending differences associated with a 1-SD increase in the corresponding measure and were interpreted within each analysis.

As the primary longitudinal analysis, we used a hybrid within-between specification based on the Mundlak approach to determine whether the association between digitalization and primary care spending reflected persistent between-county differences or within-county changes over time [23]. County-level digitalization was broken down into a between-county component, defined as the county-specific mean across all observed years, and a within-county component, defined as annual deviations from the mean. The model was specified as follows:


$$Y_{it}=\beta_{0}+\beta_{1}{\overset{-}{D}}_{i}+\beta_{2}\left(D_{it}-{\overset{-}{D}}_{i}\right)+\gamma_{t}+\varepsilon_{it}$$

In this equation, *Y_it_* is the per capita Medicare primary care spending for FQHC and RHC services in county *i* and year *t*, *D_it_* is the standardized digitalization subindex, ${\overset{-}{D}}_{i}$ is the county-specific mean of digitalization, and $\left(D_{it}-{\overset{-}{D}}_{i}\right)$ is the within-county deviation. Year fixed effects were included to account for secular trends, and SEs were clustered at the county level.

We then conducted complementary longitudinal analyses to examine nonlinearity, digitalization trajectories, and contextual heterogeneity. To assess potential nonlinearity, counties were categorized into low-, medium-, and high-digitalization groups corresponding to the tertiles of the digitalization distribution [24]. Regression models compared spending of the medium- and high-digitalization groups with the low-digitalization group, which served as the reference.

To capture dynamic patterns of digital development, we additionally conducted a trajectory analysis based on 2 county-level features derived from digitalization data from 2017 to 2023: the mean level of digitalization and its linear growth rate over time [25]. Standardized trajectory features were entered into k-means clustering. Average silhouette coefficients for solutions with 2 to 5 clusters were 0.380, 0.396, 0.404, and 0.389, respectively. A 3-cluster solution was retained based on parsimony and substantive interpretability. Spending was then compared across trajectory groups using models with year fixed effects and county-clustered SEs. Counties with persistently low digitalization and limited growth served as the reference group.

Finally, as an exploratory analysis of contextual heterogeneity, we estimated models including an interaction between standardized digitalization and baseline primary care spending [26]. Baseline spending was defined using county-level spending in 2017, the first year of the study period. These interaction analyses were interpreted descriptively because baseline spending was derived from an earlier measurement of the outcome.

Using 2023 data, we conducted a cross-sectional decomposition analysis to examine structural contributors to cross-county variation in primary care spending. This analysis was descriptive and comparative, focusing on the relative roles of digitalization, health care access, and socioeconomic conditions.

We first estimated multivariable linear regression models with primary care spending as the outcome and the 3 DHI subindexes as predictors, including pairwise interactions to assess cross-dimensional dependence. Multicollinearity among the main effects and interaction terms was assessed using variance inflation factors. To capture nonlinear and heterogeneous relationships not captured by linear regression models, we then fit gradient-boosted regression tree models (Extreme Gradient Boosting; XGBoost) using a 5-fold cross-validation framework with hyperparameters selected based on out-of-sample performance. Predictive performance was evaluated using the cross-validated coefficient of determination (*R*^2^) and mean absolute error and compared with the multivariable linear regression model and a mean-only benchmark. Model interpretability was enhanced using Shapley additive explanations (SHAP) values computed on validation data. Overall feature importance was summarized using the mean absolute SHAP value across observations, whereas summary and dependence plots were used to characterize the relative predictive contributions and context-dependent patterns of the 3 DHI dimensions [17].

Finally, to assess distributional heterogeneity, we conducted gradient boosting regression analysis for the tertiles of spending. Separate models were fit for counties in low-, medium-, and high-spending segments, and SHAP-based feature importance was compared across tertiles to assess how the roles of digitalization, health care access, and socioeconomic conditions varied across the spending distribution [27].

Year fixed effects were included to account for common annual changes in spending levels. Because per capita FQHC and RHC spending was right skewed, we conducted sensitivity analyses using log-transformed spending, defined as *ln(spending + 1)*, and spending was winsorized at the 1st and 99th percentiles. Reported zero-spending observations were retained in all analyses. The untransformed dollar outcome was retained for the primary analysis to preserve the direct interpretation of coefficients in per capita dollars. To assess the potential influence of pandemic-related changes in digitalization and service use, we repeated the primary within-between analysis after excluding observations from 2020 and 2021.

Residual spatial autocorrelation was assessed separately by year using the global Moran *I* with row-standardized queen-contiguity weights and 9999 permutations. We further conducted a spatial sensitivity analysis incorporating state fixed effects and state-clustered SEs to account for shared policy, health system, and regional characteristics among counties within the same state.

All analyses were conducted using Python (version 3.13.5; Python Software Foundation) with libraries including Matplotlib, NumPy, Pandas, scikit-learn, SHAP, statsmodels, and XGBoost. Statistical significance was assessed using 2-sided tests, and 95% CIs and *P* values were reported where applicable.

### Ethical Considerations

This study was reviewed by the University of North Carolina at Chapel Hill Office of Human Research Ethics and was determined not to constitute human subject research under applicable federal regulations. Therefore, institutional review board approval was not required (26-1879). This study used publicly available, county-level aggregate data and did not involve identifiable private information or direct interaction with individuals. Accordingly, informed consent was not applicable. No individuals were recruited or compensated for participation.

## Results

### Study Sample

The analytical sample included 20,898 county-year observations from 2993 US counties. The panel was nearly balanced, with 99.1% (2967/2993) of the counties contributing observations for all 7 study years. Table 1 summarizes descriptive statistics for county-level spending and the 3 DHI dimensions.

**Table 1.** Descriptive statistics for Medicare federally qualified health center (FQHC) and rural health clinic (RHC) spending and Digital Health Index measures across US counties (2017-2023).

Variables	Values, mean (SD)	Values, median (IQR)
Medicare FQHC and RHC primary care spending per capita (US $; 2017-2023)	186.34 (207.58)	103.95 (28.71-284.79)
Digitalization subindex—2017-2023 (0-1)	0.51 (0.28)	0.51 (0.26-0.75)
Digitalization subindex—2023 (0-1)	0.54 (0.13)	0.55 (0.46-0.64)
Health care access subindex (2023; 0-1)	0.45 (0.21)	0.44 (0.30-0.62)
Socioeconomics subindex (2023; 0-1)	0.57 (0.10)	0.57 (0.50-0.64)

### Primary Within-Between Analysis

The primary within-between analysis revealed that the association between digitalization and primary care spending was driven primarily by persistent between-county differences rather than within-county changes over time. The between-county component of digitalization was strongly negatively associated with spending (β=−67.62, 95% CI −73.51 to −61.74; *P*<.001), whereas the within-county component was not significant (β=–2.72, 95% CI −9.10 to 3.67; *P*=.40).

### Complementary Longitudinal Analyses (2017-2023)

Complementary analyses examined nonlinear patterns, digitalization trajectories, and variation in the association according to baseline spending levels.

Threshold analyses demonstrated a pronounced nonlinear relationship. Compared with counties in the lowest digitalization group, spending was lower in the middle-digitalization group (β=–35.25, 95% CI −49.59 to −20.92; *P*<.001) and substantially lower in the highest-digitalization group (β=–165.05, 95% CI −179.74 to −150.36; *P*<.001), consistent with a threshold effect concentrated at high levels of digitalization.

Trajectory analyses further indicated that counties characterized by earlier and sustained digital development had significantly lower spending than the reference group (β=–123.34, 95% CI −138.24 to −108.43; *P*<.001), whereas counties with later or more limited digital growth did not differ significantly from the reference group (β=14.00, 95% CI −4.61 to 32.62; *P*=.14).

After adjustment for baseline spending, digitalization remained negatively associated with spending (β=–13.48, 95% CI −16.00 to −10.96; *P*<.001). Baseline spending was strongly associated with subsequent spending (β=1.06, 95% CI 1.04-1.08; *P*<.001). The positive interaction between digitalization and baseline spending (β=0.049, 95% CI 0.03-0.07; *P*<.001) indicated that the inverse association between digitalization and subsequent spending was less pronounced among counties with higher baseline spending.

### Cross-Sectional Decomposition Analysis (2023)

**Figure 1.**
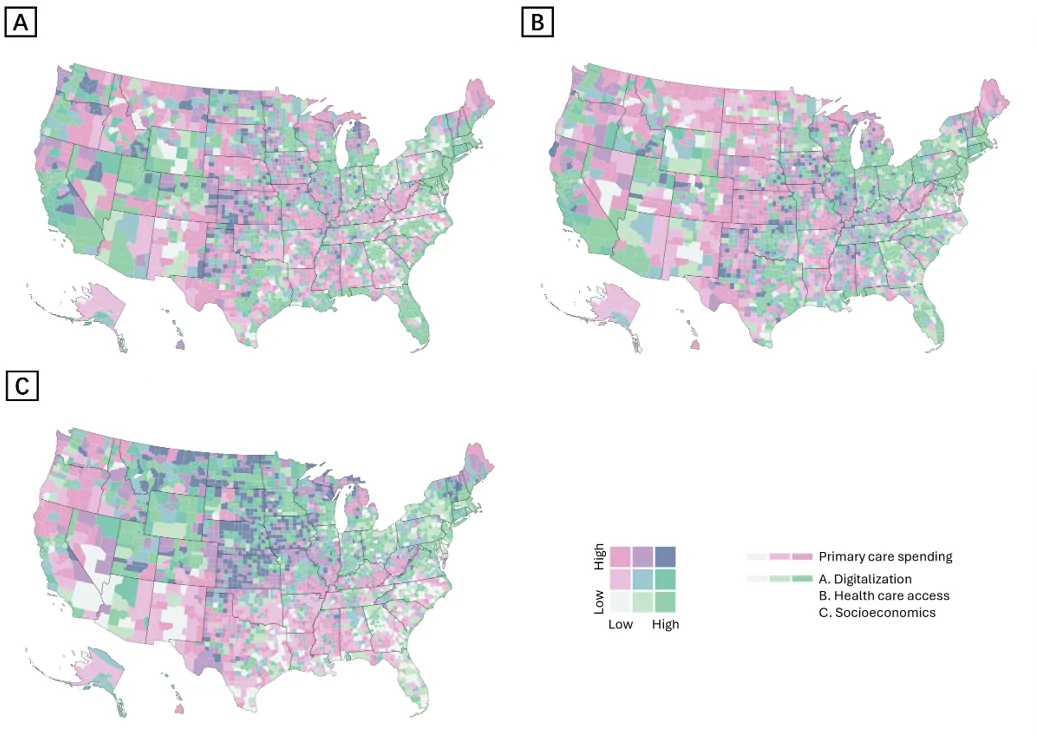
The geographic co-occurrence of county-level primary care spending with each domain of the Digital Health Index (DHI): (A) digitalization, (B) health care access, and (C) socioeconomic conditions. Counties were grouped into low, medium, and high levels for primary care spending and for each DHI domain and are displayed using bivariate color schemes to illustrate their joint spatial distribution.

Figure 1 illustrates the spatial co-occurrence of county-level primary care spending with the 3 DHI dimensions—digitalization, health care access, and socioeconomic conditions—across the United States. Distinct spatial patterns were observed across dimensions, providing descriptive context for the cross-sectional decomposition analyses.

In multivariable regression models including all 3 dimensions, health care access exhibited the strongest negative association with spending (β=–71.13, 95% CI −81.20 to −61.07; *P*<.001), followed by digitalization (β=–48.48, 95% CI −59.75 to −37.21; *P*<.001), whereas socioeconomic conditions were positively associated with spending (β=22.01, 95% CI 13.12-30.89; *P*<.001). Significant negative interactions were observed between digitalization and health care access (β=–20.02, 95% CI −28.51 to −11.53; *P*<.001) and between digitalization and socioeconomic conditions (β=–10.75, 95% CI –20.35 to –1.15; *P*=.03). Variance inflation factors ranged from 1.42 to 2.28, indicating no substantial multicollinearity among the main effects and interaction terms.

**Figure 2.**
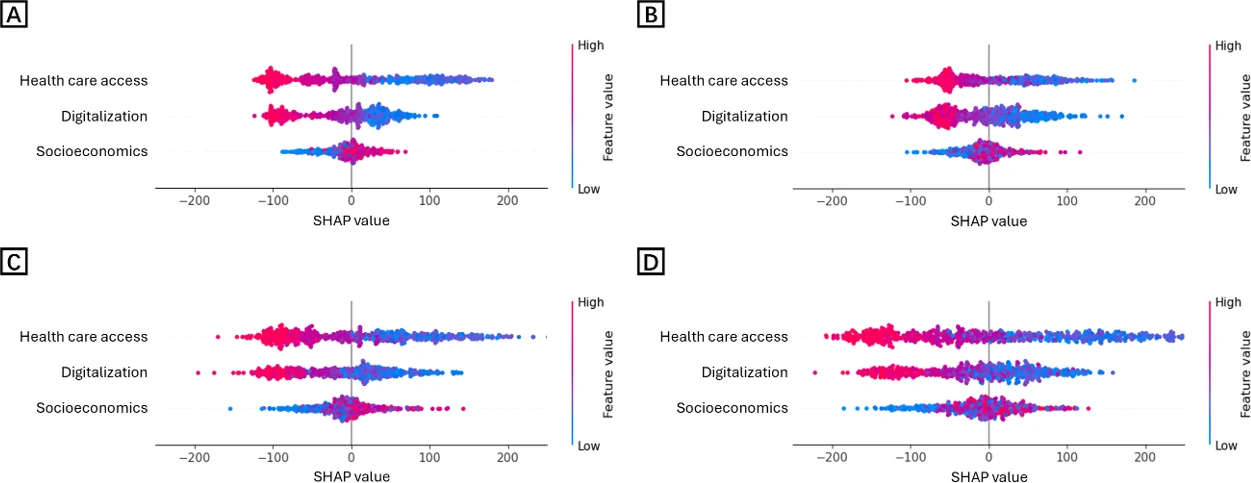
Shapley additive explanations (SHAP)–based predictive contributions of Digital Health Index dimensions to Medicare federally qualified health center and rural health clinic spending across US counties (2023). Panel A shows SHAP summary plots for the full sample, and panels B to D show SHAP summary plots stratified by low-, medium-, and high-spending areas, respectively. In each panel, points represent county-level observations; the horizontal position reflects the SHAP value, which is the contribution of a given domain to model prediction; and color indicates the domain value from lower to higher levels. Across the full sample and all spending strata, health care access showed the broadest SHAP spread and, therefore, the greatest contribution to model prediction, followed by digitalization, whereas socioeconomic conditions contributed less strongly overall.

XGBoost provided modest improvement in out-of-sample predictive performance. In 5-fold cross-validation, XGBoost showed modestly better predictive performance than linear regression. The mean cross-validated *R*^2^ was 0.213 (SD 0.042) for XGBoost and 0.208 (SD 0.027) for linear regression. XGBoost achieved a mean absolute error of US $159.13 (SD 5.77) compared with US $165.48 (SD 5.61) for linear regression and US $197.36 (SD 6.35) for the mean-only benchmark. Beyond this incremental improvement in predictive performance, SHAP summary plots identified health care access as the most informative dimension for distinguishing county-level spending, followed by digitalization, with socioeconomic conditions contributing the least (Figure 2A).

**Figure 3.**
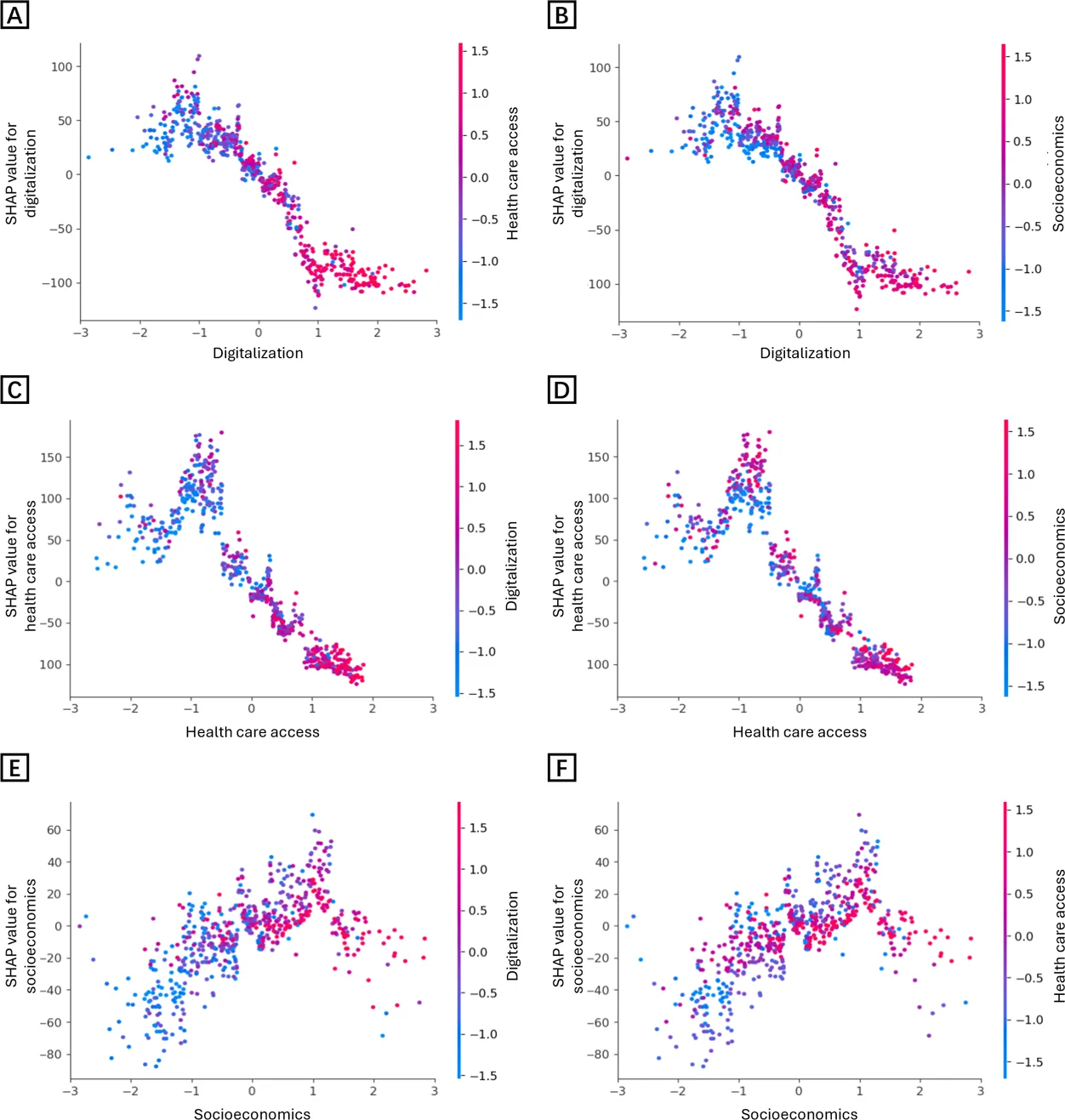
Context-dependent predictive patterns among Digital Health Index (DHI) dimensions and Medicare federally qualified health center and rural health clinic spending across US counties (2023). Panels A to F show Shapley additive explanations (SHAP) dependence plots illustrating how the association between each DHI domain and predicted primary care spending varies according to the levels of the other domains. In each panel, the x-axis shows the value of the focal domain, the y-axis shows its SHAP value (ie, the contribution of that domain to model prediction), and point color indicates the level of the accompanying contextual domain. (A) is the association between digitalization and spending across levels of health care access, (B) is the association between digitalization and spending across levels of socioeconomic conditions, (C) is the association between health care and spending across levels of digitalization, (D) is the association between health care access and spending across levels of socioeconomic conditions, (E) is the association between socioeconomic conditions and spending across levels of digitalization, and (F) is the association between socioeconomic conditions and spending across levels of health care access.

SHAP dependence plots further demonstrated contextual variation (Figure 3). Digitalization contributed more in counties with limited health care access and poorer socioeconomic conditions, whereas health care access contributed more in counties with lower digitalization and greater socioeconomic advantage. Socioeconomic conditions were more influential in counties with higher levels of digitalization and health care access.

**Figure 4.**
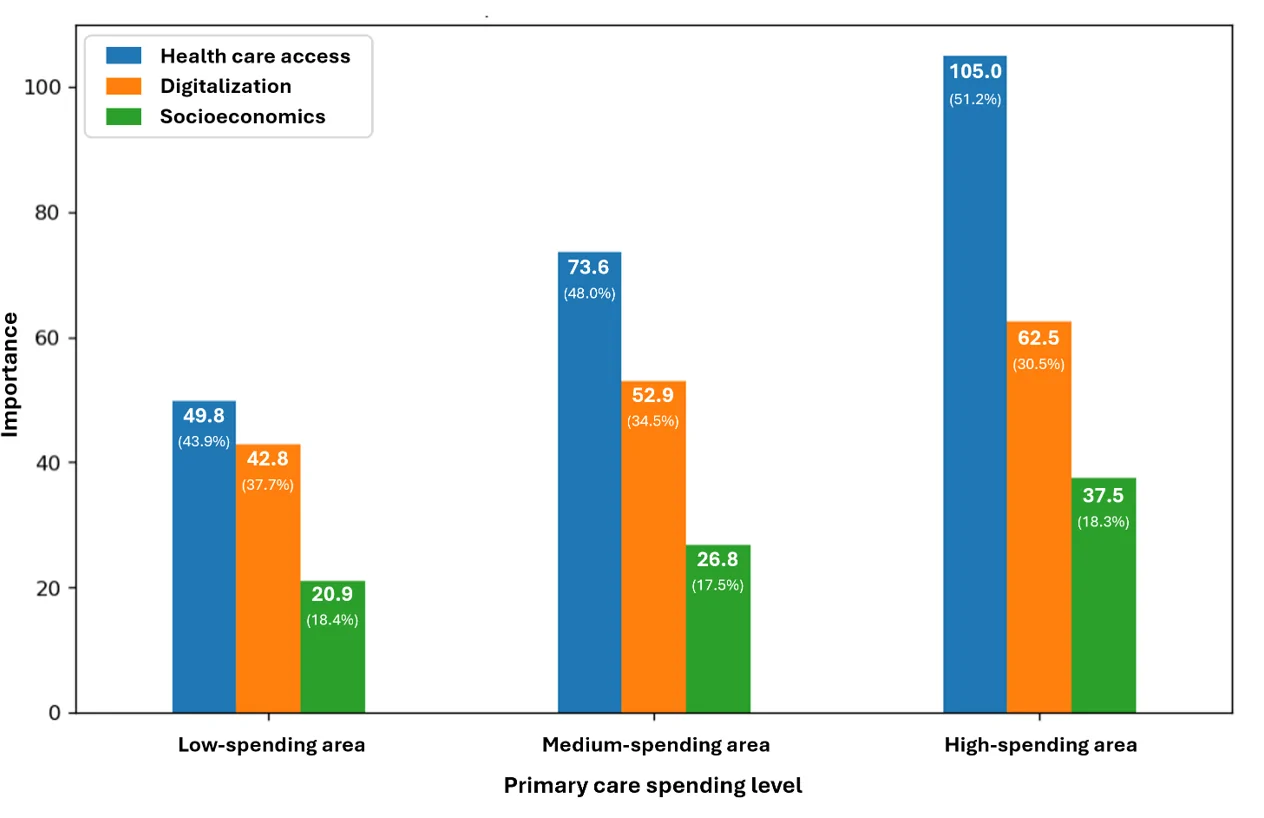
Normalized Shapley additive explanations importance shares of Digital Health Index dimensions across federally qualified health center and rural health clinic spending levels in US counties (2023).

Tertile-specific gradient boosting models indicated broadly similar patterns across the spending distribution. Across low-, medium-, and high-spending counties, health care access remained the strongest contributor to predicted spending, followed by digitalization, whereas socioeconomic conditions consistently contributed the least. When feature importance was normalized within each spending tertile, the relative contribution of health care access increased from 43.9% in low-spending counties to 51.2% in high-spending counties, whereas the contribution of digitalization decreased from 37.7% to 30.5%. The relative contribution of socioeconomic conditions remained comparatively stable (Figures 2B-2D and Figure 4).

### Sensitivity Analysis

Sensitivity analyses yielded findings consistent with the primary specification. Using log-transformed spending, the between-county association remained inverse and statistically significant (β=–0.71, 95% CI −0.75 to −0.67; *P*<.001), whereas the within-county association remained nonsignificant (β=–0.02, 95% CI −0.05 to 0.02; *P*=.38). Results were similar after winsorizing spending at the 1st and 99th percentiles, with a significant between-county association (β=–67.51, 95% CI −73.21 to −61.81; *P*<.001) and a nonsignificant within-county association (β=–3.62, 95% CI −9.62 to 2.38; *P*=.24). The primary findings were also robust to exclusion of the acute COVID-19 pandemic period. After excluding 2020 and 2021, the between-county association remained inverse and statistically significant (β=–70.03, 95% CI −76.03 to −64.02; *P*<.001), whereas the within-county association remained nonsignificant (β=–4.99, 95% CI −12.58 to 2.59; *P*=.20).

Residual spatial autocorrelation remained detectable after accounting for state-level differences, with the global Moran *I* ranging from 0.256 to 0.318 across study years (permutation *P*<.001 for all years). In the sensitivity analysis incorporating state fixed effects and state-clustered SEs, the between-county association remained inverse and statistically significant (β=–84.66, 95% CI −99.22 to −70.09; *P*<.001), whereas the within-county association remained nonsignificant (β=–2.72, 95% CI −10.74 to 5.31; *P*=.51).

## Discussion

### Principal Findings

In this study, county-level digitalization was consistently associated with lower Medicare primary care spending for FQHC and RHC services across multiple analytic approaches. The within-county estimate was small and nonsignificant, indicating that changes in county digitalization were not associated with corresponding changes in spending during the study period. The observed inverse association was therefore concentrated in long-standing differences between counties. Digitalization may reflect accumulated technological, organizational, and health system capacity associated with how primary care services are organized and used. The meaning of lower spending may vary across local contexts, particularly in safety net settings where service availability and unmet need also shape patterns of use.

The longitudinal between-within analysis showed that counties with persistently higher levels of digitalization had substantially lower Medicare spending for FQHC and RHC services, whereas year-to-year within-county changes in digitalization relative to the common study period reference were not significantly associated with changes in spending. This pattern suggests that digitalization functions more as a structural feature of local primary care systems than as a short-term policy lever capable of producing immediate changes in spending. These findings are consistent with the view that digital health infrastructure may provide an enabling environment for digitally supported care, while the adoption and integration of technologies such as electronic health records, health information exchange, and telehealth capacity require sustained investment, organizational learning, and workflow redesign before it can meaningfully influence resource use in primary care settings [28]. In FQHC and RHC settings, where organizational and operational constraints are often substantial, these processes are unlikely to unfold rapidly and may therefore not be captured by short-term changes in digital capacity [29].

The nonlinear and threshold analyses suggest that the association between digitalization and primary care spending depends not simply on whether digital tools are adopted but on the extent to which digital capacity has matured and become integrated within local primary care systems. Spending reductions were modest at intermediate levels of digitalization and substantially larger only among counties with high digitalization levels, indicating a threshold effect. This pattern suggests that incremental or fragmented adoption may be insufficient to produce meaningful changes in care coordination, visit substitution, or population management in FQHC and RHC settings [28,29]. Instead, spending-related benefits may be more likely to emerge when digitalization is implemented broadly and sustained over time.

Trajectory-based analyses further underscore that the association between digitalization and primary care spending depends not only on the level of digitalization achieved but also on the pathway through which counties reach it. Counties characterized by early and sustained digital development had significantly lower primary care spending, whereas counties with later or more limited digital growth did not differ meaningfully from counties with persistently low levels of digitalization. This finding highlights the importance of cumulative experience and institutional adaptation. Early digital adoption may provide primary care organizations with more time to redesign care processes, integrate digital tools into chronic disease management, and establish organizational capabilities that translate into more efficient use of primary care resources [30,31].

The effect modification analysis indicates that while digitalization was associated with lower primary care spending overall, its association was weaker in counties with higher baseline spending. This finding suggests that the role of digitalization varies according to underlying spending conditions and may be less pronounced where structural drivers of service use are more substantial, although the use of an earlier spending measurement as the effect modifier also introduces the possibility of outcome persistence and regression to the mean. Elevated disease burden, workforce shortages, access constraints, and local care delivery patterns may continue to shape spending even in counties with stronger digital capacity [11,32,33]. In high-spending settings, digital capacity alone is unlikely to reverse entrenched use patterns without complementary reforms in care delivery models, workforce capacity, or payment incentives.

The separate 2023 cross-sectional decomposition analysis highlights the distinct and interacting contributions of digitalization, health care access, and socioeconomic conditions to primary care spending. Health care access was the strongest independent correlate of lower Medicare FQHC and RHC spending, followed by digitalization, while socioeconomic conditions were positively associated with spending, possibly reflecting greater realized use of services. Digitalization also interacted with both health care access and socioeconomic conditions. The spending-reducing association of digitalization was strongest in counties with better access and more favorable socioeconomic conditions, suggesting that digital infrastructure is most effective when layered onto existing primary care capacity rather than deployed in its absence [34].

Across the spending distribution, health care access remained the most informative dimension for distinguishing counties, followed by digitalization, whereas socioeconomic conditions had a more limited direct role. As spending increased, health care access became an increasingly dominant constraint, while the relative contribution of digitalization declined and that of socioeconomic conditions remained comparatively stable. These findings suggest that digitalization exerts a persistent but secondary influence across spending levels, whereas access constraints become especially salient in higher-spending primary care settings [11]. SHAP-based analyses further suggest that digitalization and health care access may partially compensate for one another in structurally disadvantaged contexts, highlighting the function of digitalization and health care access as adaptive mechanisms in resource-constrained primary care systems [28].

From a policy perspective, these findings provide relevant context for Centers for Medicare & Medicaid Services (CMS) initiatives that link digital infrastructure with broader primary care reform. Recent CMS actions, including advanced primary care management services, the Achieving Healthcare Efficiency Through Accountable Design model, and CMS’s interoperability framework, illustrate policy approaches that combine digitalization with payment reform, care coordination, and stronger practice infrastructure [35-37]. Although these initiatives were not evaluated in the present study, their integrated approach is consistent with the observed role of digitalization as part of a broader local health system context. In high-demand or high-spending counties, digitalization alone is unlikely to offset entrenched access barriers, workforce shortages, or other structural constraints. Early and sustained digital investment sequenced alongside broader reforms in access, workforce capacity, and primary care delivery models may support the development of stronger primary care systems [38]. Evaluating such investments will require attention to service use, access, and quality and patient outcomes alongside spending. Ensuring that these gains reach medically underserved and rural populations—where the gap between digital capacity and unmet need is widest—should be a central objective of Medicare primary care policy.

This study had several limitations. First, the observational design precludes causal inference, and reverse causation and residual confounding cannot be excluded. Because the primary association was concentrated in persistent between-county differences, it may partly reflect relatively stable differences in rurality, health system capacity, workforce availability, socioeconomic resources, population health needs, or regional and state policy contexts. The within-between design distinguished structural differences from temporal changes but did not eliminate confounding within the between-county component. Second, digitalization and other structural characteristics were measured at the county level and may not fully capture within-county heterogeneity or variation across individual primary care organizations; future work linking organizational-level digital capacity to spending would strengthen these findings. Third, Medicare spending for FQHC and RHC services reflects a specific segment of primary care and may not generalize to other payers or care settings. Spending was not examined alongside corresponding measures of service volume, access, or quality or patient outcomes. We therefore could not determine whether lower spending reflected more efficient resource use, lower service use, or unmet primary care needs. Fourth, machine learning analyses were intended to characterize predictive heterogeneity rather than estimate interpretable parameters. These analyses should therefore be viewed as complementary to rather than substitutes for regression-based findings.

### Conclusions

Higher county-level digitalization was associated with lower Medicare primary care spending for FQHC and RHC settings, with the association concentrated in persistent differences between counties. Within-county changes in digitalization were not associated with corresponding changes in spending during the study period. The findings identified digitalization as a structural correlate of spending and a potential marker of local technology, organizational, and health system capacity. Its role in primary care is therefore best considered within the broader context of health care access, workforce capacity, care coordination, and supportive payment and delivery systems. Further research using longer follow-up, organization-level measures, and causal or quasi-experimental designs is needed to determine whether improvements in digital capacity lead to changes in primary care spending.

## Abbreviations

CMS: Centers for Medicare & Medicaid Services
DHI: Digital Health Index
FQHC: federally qualified health center
RHC: rural health clinic
SHAP: Shapley additive explanations
XGBoost: Extreme Gradient Boosting
